# PD94 Adapting Existing Evidence-Based Clinical Practice Guidelines: A Case Example with Sexually Transmitted Infections Guidelines In Catalonia, Spain

**DOI:** 10.1017/S0266462324003416

**Published:** 2025-01-07

**Authors:** Maria Dolors Estrada Sabadell, Johanna Milena Caro Mendivelso, Anna Godo Pla, Rosa Maria Vivanco-Hidalgo, Caridad Almazán Sáez, Paula Lletjós Botey, Rosa Mansilla Lou, Mercè Armelles Sebastià, Mireia Alberny Iglesias

## Abstract

**Introduction:**

In the field of sexually transmitted infections (STI), high quality clinical practice guidelines (CPGs) have already been developed using rigorous methodology. To avoid duplicating effort, make cost-effective use of available resources, and facilitate customization to reflect local contexts, we adapted a CPG for prevention, care, and control of STIs in Catalonia, Spain.

**Methods:**

The Guideline Adaptation Group (GAG) used the ADAPTE framework to guide transcontextual adaptation. The ADAPTE model has three phases: set-up; adaptation, with five main criteria for determining the relevance of a CPG and the adaptability of its recommendations (quality, currency, content, consistency, and acceptability or applicability); and finalization. We used the Spanish version of the AGREE II instrument to rate and select appropriate CPGs and the RIGHT-AD@pt checklist to guide reporting of the adaptation process. Each recommendation was given a level of evidence and a grade using the GRADE framework.

**Results:**

Once the feasibility of carrying out the adaptation was confirmed, the governance structure was defined, which comprised a steering committee (n=8 members) and an expert committee (n=93 members). Clinical questions were defined from 164 recommendations taken from a CPG published in 2009. The systematic search identified 759 records published from 2016 to 2023. Of these, 57 CPGs met the inclusion criteria. On average, items 10 and 12 of the AGREE II scored between five and seven points (moderate to high quality methodological rigor). Content and consistency are being analyzed and matrices are being prepared to assess acceptability and applicability.

**Conclusions:**

The ADAPTE methodology provides structure, rigor, and efficiency to the transcontextual adaptation of CPG recommendations. We expect that its application will provide practical and timely recommendations on the prevention, care, and control of STIs in the Catalan context.

